# An Experimental Study on Micro-Milling of a Medical Grade Co-Cr-Mo Alloy Produced by Selective Laser Melting

**DOI:** 10.3390/ma12132208

**Published:** 2019-07-09

**Authors:** Gabriele Allegri, Alessandro Colpani, Paola Serena Ginestra, Aldo Attanasio

**Affiliations:** Department of Mechanical and Industrial Engineering, University of Brescia, Via Branze 38, 25123 Brescia (BS), Italy

**Keywords:** Additive Manufacturing, micro-milling, Selective Laser Melting, cobalt-chromium-molybdenum alloys, ASTM F75

## Abstract

Cobalt-chromium-molybdenum (Co-Cr-Mo) alloys are very promising materials, in particular, in the biomedical field where their unique properties of biocompatibility and wear resistance can be exploited for surgery applications, prostheses, and many other medical devices. While Additive Manufacturing is a key technology in this field, micro-milling can be used for the creation of micro-scale details on the printed parts, not obtainable with Additive Manufacturing techniques. In particular, there is a lack of scientific research in the field of the fundamental material removal mechanisms involving micro-milling of Co-Cr-Mo alloys. Therefore, this paper presents a micro-milling characterization of Co-Cr-Mo samples produced by Additive Manufacturing with the Selective Laser Melting (SLM) technique. In particular, microchannels with different depths were made in order to evaluate the material behavior, including the chip formation mechanism, in micro-milling. In addition, the resulting surface roughness (Ra and Sa) and hardness were analyzed. Finally, the cutting forces were acquired and analyzed in order to ascertain the minimum uncut chip thickness for the material. The results of the characterization studies can be used as a basis for the identification of a machining window for micro-milling of biomedical grade cobalt-chromium-molybdenum (Co-Cr-Mo) alloys.

## 1. Introduction

Additive Manufacturing (AM) is a technology based on a layer-by-layer fabrication strategy that allows the build-up of a complex part with remarkable design flexibility [1]. The main characteristic that distinguishes AM systems when compared to conventional manufacturing technologies is the great potential in producing customized parts for several applications [2]. In particular, biomedical parts can be specifically designed for the patient, customizing the component in order to address specific biomedical needs. AM also allows the production of devices with a shape suitable for complex anatomical regions of the human body [3]. Biomedical devices, designed according to patient-specific needs, enhance the outcomes of the biomedical implant and guarantee better integration with the human body [4]. Therefore, the study of the technological processes that can be used for medical devices production is a necessity and a research challenge, in particular, for the identification of an optimized process chain for these applications [5].

**Figure 1:**
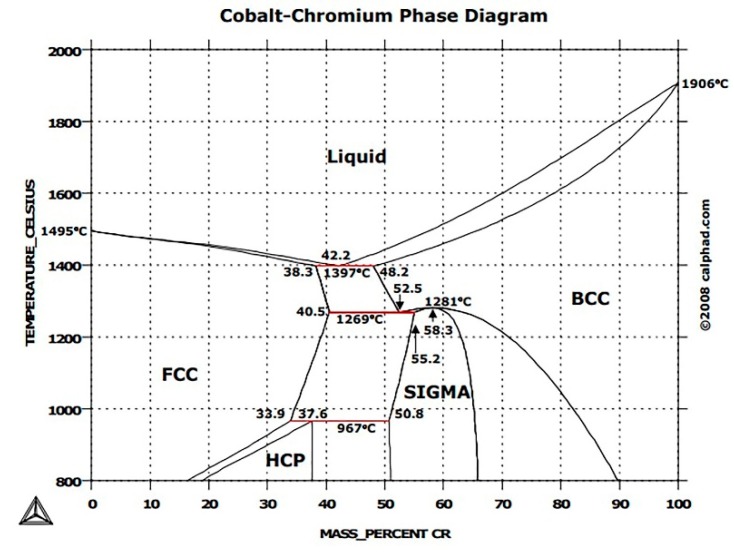
CoCr alloy equilibrium diagram with the microstructural changes (under thermodynamic equilibrium conditions) [10].

ASTM F75 is a non-magnetic cobalt-chromium-molybdenum (Co-Cr-Mo) alloy, exhibiting high strength, corrosion, and wear resistance, as well as excellent biocompatibility. CoCr alloys were first synthesized in 1907 by Haynes [6] who observed the low chemical reactivity and the high strength that characterizes this basic binary alloy. Furthermore, it was found that adding Molybdenum (Mo) or Tungsten (W) results in a remarkable increase in strength [7]. Figure 1 shows the equilibrium diagram for binary Co-Cr alloy. The ϒ-phase is characterized by a face-centered-cubic (FCC) crystal structure that provides high work hardening rates and strength [8]. Furthermore, FCC structure, absorbing the stress, can transform into the more stable hexagonal-close-packed (HCP) structure (ε-phase) [9].

In the biomedical implants field, medical grade cobalt-chromium alloys show all the required properties for class III medical devices [6]. These materials are suitable for implantable medical device components (in particular, orthopedic implants [11]) due to their mechanical and physical properties [12] that include high strength, stiffness, hardness, wear resistance, and excellent biocompatibility related to the formation of a hard passive surface oxide [13]. Nowadays, the market demand for medical implants has led to an increased demand for these alloys [14]. 

Other applications for these materials are related to the aerospace and energy fields, where cobalt alloys play an important role in gas turbines performances. While vacuum cast nickel alloys predominate in the hot sections of modern aero-turbine engines, cobalt alloys are used for particularly demanding applications, such as fuel nozzles and vanes, for industrial gas turbines [15].

Co-Cr-Mo components are commonly produced by casting or forging processes [16]. Importantly, these processes affect the functional surface characteristics and device performance when high levels of tribomechanical stress are involved during the component life cycle [17]. Furthermore, these processes produce near-net shape forms, so finishing operations are necessary in order to obtain the desired final component [18].

Focusing on the biomedical applications, AM technology can be actually exploited in the process chain of medical device fabrication for the production of complex components with a high level of flexibility. However, AM has some disadvantages, in particular, when high precision is required or 3D printed parts need functional micro-scale features. Therefore, even if components are realized by AM, micromachining can be exploited in this process chain to produce specific features and to improve the final part characteristics, such as roughness or surface mechanical properties [19]. Despite the fact that high precision machining allows obtaining the required shapes and dimensions, the geometry, dimensional tolerances, surface finish, and integrity need to be achieved cost-effectively [20,21].

The importance that mechanical cutting processes could have in this process chain due to the improvement in the characteristics of the final part is remarkable [22]. However, limited research has been reported about the basic mechanisms involved in the cutting of any class of Co-Cr-Mo alloy. Therefore, this paper tries to introduce fundamental knowledge on the cutting mechanisms of these materials, in particular, focusing on the micro-milling process for ASTM F75. Furthermore, in order to test the behavior of this material in micro-milling produced by AM, Co-Cr-Mo samples used for this research were produced using the Selective Laser Melting (SLM) technique.

Micro-milling preliminary tests were performed to understand if an SLM ASTM F75 sample exhibits a layered effect. These tests analyzed microchannels produced on the SLM sample at different axial depths of cut to verify if a change in the forces occurred when channels were made at increased depth. Moreover, the paper presents an analysis of the cutting force components produced during the micro-milling of the SLM ASTM F75 with the aim of determining the minimum uncut chip thickness (MUCT).

Furthermore, the machined sample was analyzed with a laser profilometer for profiles acquisition and roughness (Ra) measurements [23]. Finally, the microhardness of the machined surfaces was evaluated and analyzed.

## 2. Materials and Methods 

The Co-Cr-Mo metal powder (see also Figure S1, Figure S2) used for the SLM building follows the requirements indicated in the ASTM F75 standard. This material exhibits a range of properties (strength, hardness even at high temperature, wear and corrosion resistance, high elastic modulus), summarized in Table 1, that qualify ASTM F75 for use in medical implants (orthopedic, in particular). 

For this work, the Co-Cr-Mo (ASTM F75) sample was fabricated using a Tongtai AM-250 with a laser power of 45 W (focus diameter of 50–150 μm), a scanning speed of 160 mm/s, and a layer thickness of 30 μm. The orientation of the built part was set along the XY axis (flat in relation to the building plate) [24]. The diameter of metallic particles was equal to 32.7 ± 12.6 μm, while the sample was a thin slab with dimensions of 40 mm × 20 mm × 2 mm.

**Figure 2:**
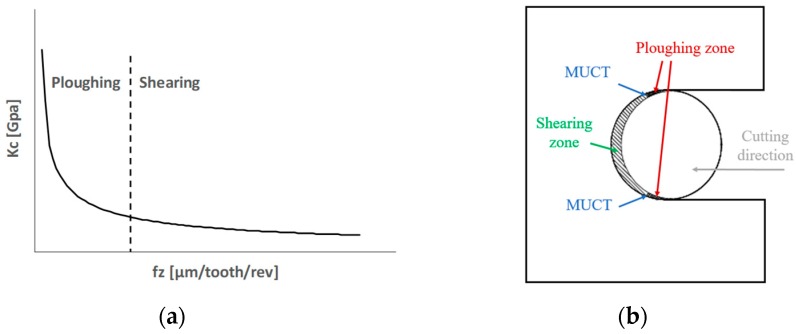
Minimum Uncut Chip Thickness (MUCT): (**a**) relation between specific cutting force and feed per tooth; (**b**) plowing and shearing in one tool revolution. Kc: specific cutting force; fz: feed per tooth.

In this work, microchannels were milled on the SLM part at a different feed per tooth values. The size effect is observed through the asymptotic growth of the specific cutting force when the feed per tooth decreases [25]. Figure 2a shows schematically the relationship between the specific cutting force (Kc) and the feed per tooth (fz). During a tool revolution, the removed material thickness ranged from zero to the feed per tooth value and then gradually returned to zero (Figure 2b). When the feed per tooth is smaller than the MUCT (plowing condition), the material is not actually cut, but it is plastically deformed. Therefore, in this condition, there is no chip formation. Furthermore, there is a strong elastic recovery, which causes an increase in the specific cutting force. When the feed per tooth is greater than the MUCT (shearing condition), the tool is actually cutting the workpiece and a chip is formed [26]. Usually, during a micro-milling operation, plowing dominant areas are located at the beginning and the end of the flute path (i.e., when the flute is getting into the workpiece and when it is exiting), while the remaining area is characterized by the shearing condition (Figure 2b). MUCT can be determined from the specific cutting force trend and, in particular, from the value that corresponds to the exponential increase of this parameter when the feed per tooth decreases (Figure 2a) [25].

## 3. Experimental Tests

First, a preliminary analysis was carried out on the SLM sample before micro-milling the channels. The aim of these tests was to determine the MUCT, also considering other quantities as roughness (Ra and Sa) and hardness and evaluating the chip morphology.

### 3.1. Preliminary Tests

**Figure 3:**
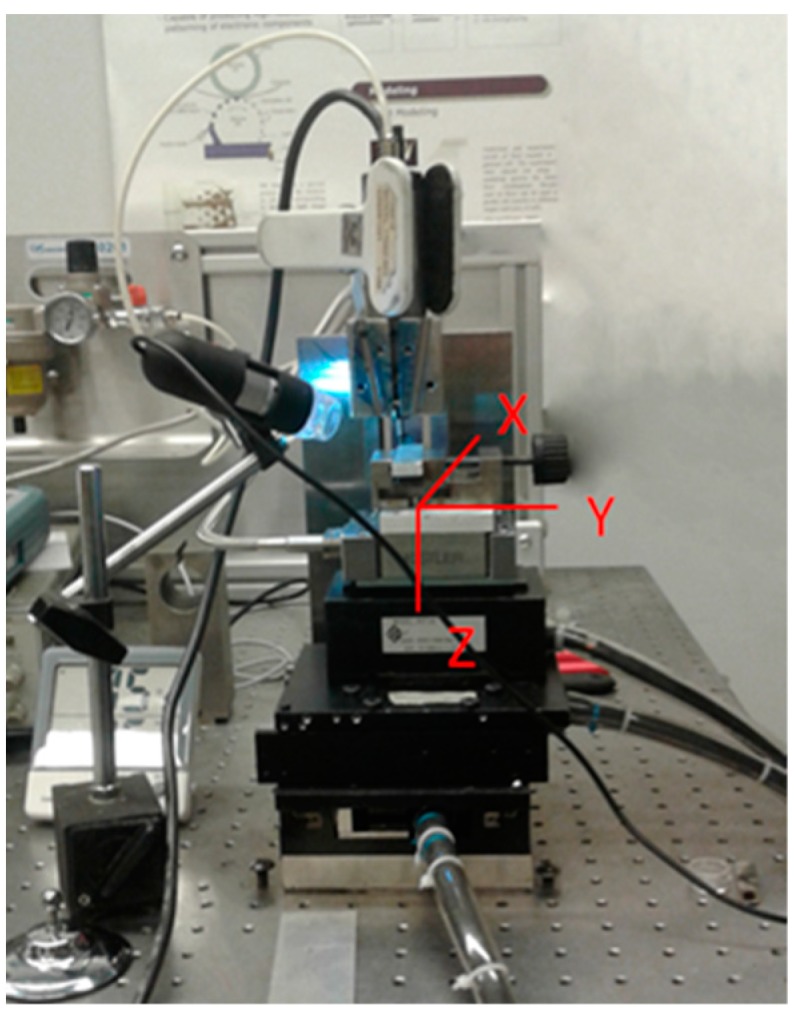
The three-axis milling machine used for the preliminary tests.

Preliminary tests were conducted in order to verify if the SLM sample exhibits a layered effect. Therefore, microchannels were produced at different axial depths of cut in order to evaluate the material behavior during the cutting process. Microchannels were made on an experimental three-axis micro milling machine, shown in Figure 3. The high-speed electric spindle that rotates from 5,000 rpm to 80,000 rpm is connected to a spindle holder made of Invar in order to minimize thermal dilatation during experiments. In order to reduce the influence of external vibration on process accuracy and measurements results, the micro-milling machine is placed on a pneumatic table. The workpiece was connected to a Kistler load cell for force measurement and to a micro-positioning stage, which allows positioning in the X, Y, and Z directions (oriented, as shown in Figure 3) with an operating stroke of 30 mm × 30 mm × 10 mm through G-Code commands. A USB micro-camera (Celestron, Torrance, California) was used to precisely monitor the micro-milling operation. A 2-flute square-end micro end mill (PMT TR-2-0020-S, Performance Micro Tool, Janesville, Wisconsin) was used in these preliminary experiments. Table 2 summarizes the main characteristics of the tool used.

**Figure 4:**
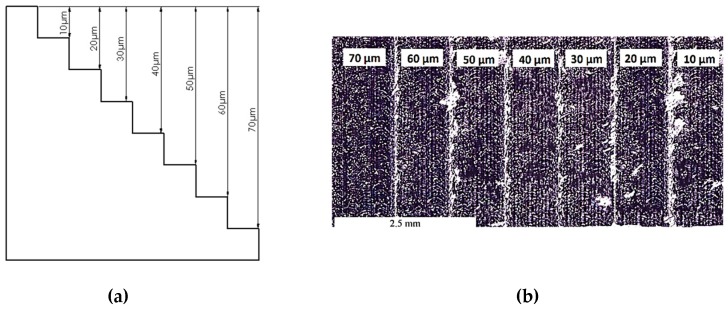
Microchannels for the layered effect test: (**a**) test design; (**b**) microchannels image after the machining.

The grain size of the metallic powder used in the SLM sample production is about 30 μm. Therefore, the depth of cut was varied from 10 µm to 70 µm with a 10 µm step to evaluate the possible differences between the micro-milling tests performed on an SLM layer and in between layers. After a preliminary face milling operation, microchannels were made, as shown in Figure 4 (see also Figure S3). Cutting speed was fixed at 25.5 mm/min (corresponding to a spindle rotation speed of 16000 rpm), and the feed per tooth was 4 μm/tooth/rev.

### 3.2. Force Measurement and MUCT Evaluation

After the preliminary investigation of the layered effect, the work focused on the machinability of this alloy in micro-milling, with the main purpose to determine the MUCT. The nano precision machine tool (KERN Pyramid Nano, Kern Micro, Eschenlohe, Germany) was used for the production of 14 microchannels with a length of 5 mm on a sample measuring 40 mm × 20 mm × 2 mm. A preliminary face milling operation was performed through five identical consecutive passes with a depth of cut of 100 μm for each step in order to obtain a complete flat worked surface. The feed per tooth (fz) was different for each channel (values ranged from 1 µm/tooth/rev to 10 µm/tooth/rev), while the depth of cut (a_p_) was kept constant at 10 µm. The cutting speed was 27.3 m/min. The tool used in this operation was a SECO 905004-MEGA-T (Table 3). The tool wear had been measured with a digital optical microscope, and the result was negligible. Between two consecutive tests, the tool had been properly cleaned to remove any stick material, as confirmed by the optical microscope observations.

**Figure 5:**
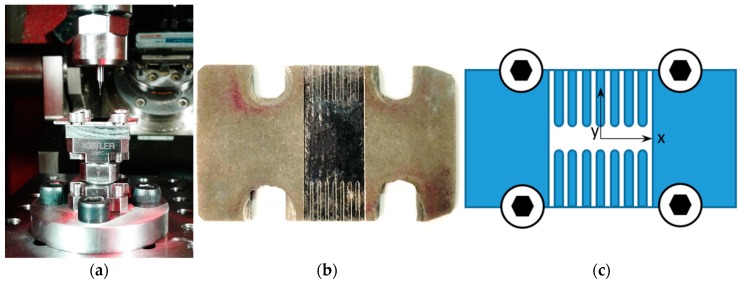
Experimental configuration: (**a**) sample fixed in the micro-milling machine; (**b**) machined microchannels; (**c**) scheme of the micro-milling tests.

The cutting forces were measured using the KISTLER 9317C load cell connected to a three-channel charge amplifier (KISTLER Type 5015A, Kistler Group, Winterthur, Switzerland) able to convert the charge signal generated by the cell in a ±10 V voltage signal. This voltage signal was acquired using a NI 9205 module mounted in a NI compact DAQ-9184 data acquisition module and exported for analysis into the LABVIEW 2018 software (National Instrument, Austin, Texas). Hammer impact test was done to correctly define the bandwidth of this force sensor, that ranges from 100 Hz to 3000 Hz [27,28]. Being the cutting force signal frequency equal to 724 Hz, the utilized system is adequate. For each microchannel, the measured forces were analyzed by considering a signal window that corresponds to 20 spindle full rotations in the steady-state zone. Figure 5a shows the sample screwed on the load cell, Figure 5b shows the 14 realized microchannels, while Figure 5c presents a scheme of the performed operations. Table 4 summarizes the different fz levels used for the microchannel tests.

### 3.3. Surface and Chip Analysis

In order to test how the roughness is affected by the process parameters, the profile and surface roughness (Ra and Sa, respectively) of the microchannels were acquired with a Mitaka PF 60 and measured using Mountains Map 8 software (Digital Surf, Besançon, France). This instrument is certificated with the GPS (geometrical product specification) ISO-25178. Ra was computed from a 4.5 mm profile measured along the microchannels with a resolution (a distance of acquisition between two consecutive points along the profile) equal to 0.1 μm. The Sa for each microchannel was computed from a measured surface of 400 μm × 300 μm that was acquired with a resolution equal to 0.1 μm in both the X and Y directions. The roughness was evaluated after removing the form error both for the profiles and the surfaces acquired. Measurements performed on the microchannels were made also on the raw material, and the results were compared.

Furthermore, the chip morphology was monitored. In particular, after each microchannel was machined, chips were collected and imaged in a Hirox RH-2000 digital microscope (Hirox, Tokio, Japan) for the morphology analysis and dimensional measurement.

### 3.4. Microhardness

Microhardness was measured in order to have additional information on the influence of the feed per tooth on the finished surface. Therefore, hardness was measured on the machined surface. In particular, using a Mitutoyo HM-200 (Mitutoyo, Sakado, Japan), 20 points on each microchannel were measured (with a distance of 200 µm between each measurement) in order to calculate the average microhardness value and the standard deviation for each channel.

## 4. Results and Discussion

### 4.1. Preliminary Tests

**Figure 6:**
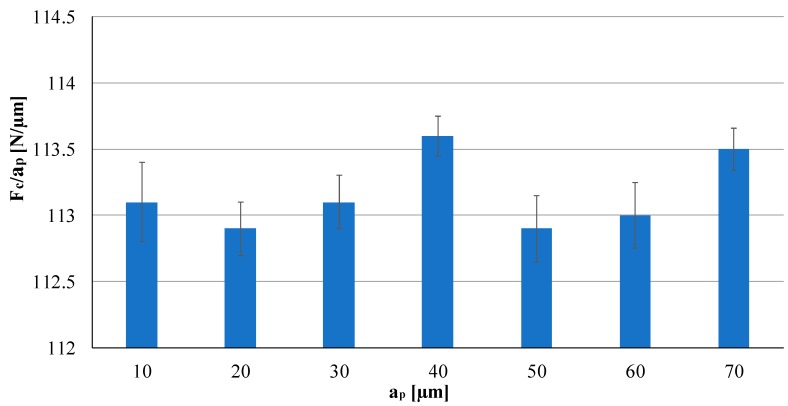
Force analysis for the layered effect test.

As regards to the layered effect investigations, the experiment analyzed the force behavior to highlight a possible variation caused by the additive layering manufacturing of the SLM part. Figure 6 presents the results of this analysis, in particular, showing the cutting force (Fc) value for each microchannel. Increasing the depth of the microchannel from 10 μm to 70 μm forces values was found to be quite similar at each level, demonstrating there is no relevant layered effect.

### 4.2. Force Measurement and MUCT Evaluation

**Figure 7:**
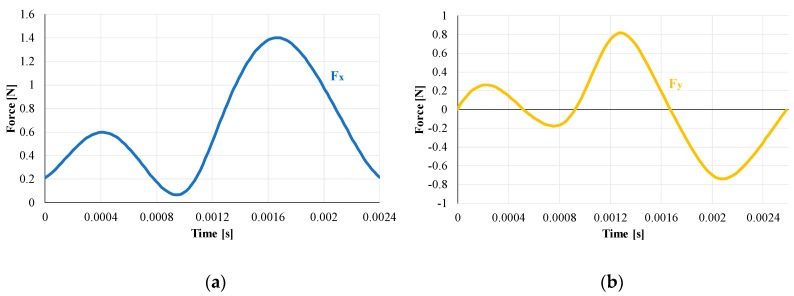

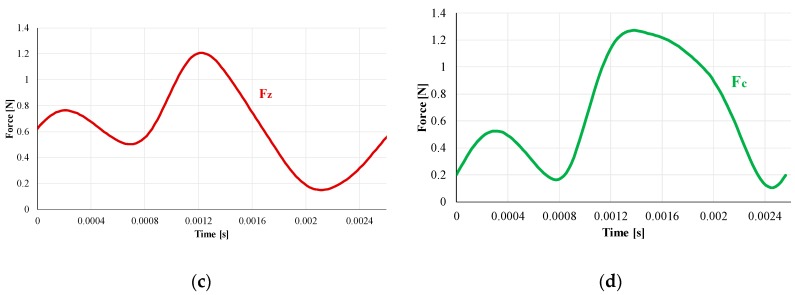
Microchannel A (fz = 10 µm)—Forces in a single spindle rotation: (**a**) Fx; (**b**) Fy; (**c**) Fz and (**d**) Fc (cutting force). fz: feed per tooth; Fx: force component along X axis; Fy: force component along Y axis; Fz: force component along Z axis.

Forces values calculated for each microchannel were analyzed for the MUCT determination. Signals were filtered with a low pass filter of 800 Hz, and a window that includes 20 full rotations of the spindle was used for the analysis. Figure 7 shows the signals that represent the forces generated during a single rotation in the first test (fz = 10 µm). In these figures, the tool runout can be clearly observed, and signals that correspond to flute 1 and 2 are recognizable in the graphs. 

**Figure 8:**
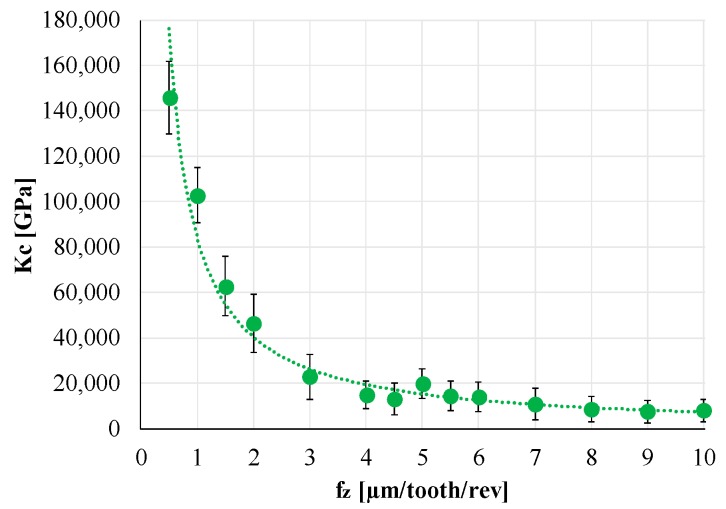
Specific cutting force (Kc).

Data analysis was then focused on the MUCT calculation. Therefore, the specific cutting force was calculated in each direction (X, Y, and Z), normalizing the force signal by fz and a_p_, and then related to fz. The same analysis was performed on the cutting force Fc. Equations (1) and (2) report formulas for Fc and Kc, while Figure 8 shows the results of the analysis for Fc.

(1) $$F_{c}=\sqrt{F_{x}^{2}+F_{y}^{2}+F_{z}^{2}}$$

(2) $$K_{c}=\frac{F_{c}}{a_{p}f_{z}}$$

Figure 8 clearly presents the specific cutting force trend. When fz decreased, Kc exponentially increased, and the graph could be divided into two regions. In particular, the plowing region was characterized by high specific cutting force values that significantly decrease in the shearing region. In fact, for low fz values, plowing conditions occurred, while increasing the feed per tooth, the cutting condition was dominated by shearing that involves the main part of the micro-milling operation. Therefore, the MUCT value could be identified in the fz interval that characterizes the transition from plowing to shearing. In particular, when fz ranged between 2 and 3 µm/tooth/rev, the slope of the Kc curve drastically reduced, so the MUCT value for this Co-Cr-Mo alloy was between 2 µm and 3 µm. The identified MUCT corresponded to 40% of the tool edge radius (r_ε_) value (equal to 5 µm).

### 4.3. Surface and Chip Analysis

**Figure 9:**
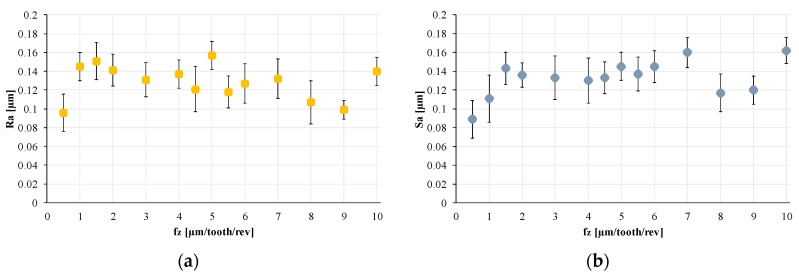
Roughness behavior when fz increases: (**a**) Ra (profile roughness) and (**b**) Sa (surface roughness).

Surface analysis—For each microchannel, the profile roughness Ra and the surface roughness Sa were measured with the aim of investigating if fz affects the microchannels’ roughness. Figure 9 shows the measurement results.

Results show that lower surface roughness can be achieved with the micro-milling operation, in particular, when the obtained roughness is compared with the raw material roughness (Ra = 11.628 µm and Sa = 11.885 µm). Furthermore, no correlation between roughness (both Ra and Sa) was present, as Figure 9 shows. In fact, no particular trend for the roughness values could be observed with an increase in fz. This result is probably due to the fact that the investigation was conducted on the roughness of the bottom of the microchannels, which is less affected by the fz value than the roughness of the microchannels’ lateral surfaces.

**Figure 10:**
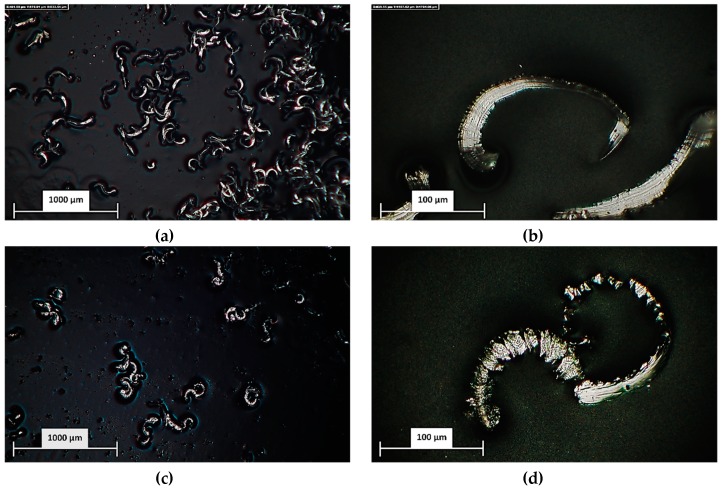
Chip analysis: (**a**) chip formation fz = 0.5 µm/tooth/rev, global view—magnification equal to 100; (**b**) chip analysis fz = 0.5 µm/tooth/rev—magnification equal to 1000; (**c**) chip formation fz = 10 µm/tooth/rev, global view—magnification equal to 100; (**d**) chip analysis fz = 10 µm/tooth/rev—magnification equal to 1000. fz: feed per tooth.

Chip analysis—The chip was collected during the machining operations and analyzed with Hirox-Rh 2000 digital microscope. In order to evaluate the chip morphology, two multifocal images were acquired for a global observation (magnification equal to 100, Figure 10a,c) and for chip inspection (magnification equal to 1000, Figure 10b,d).

**Figure 11:**
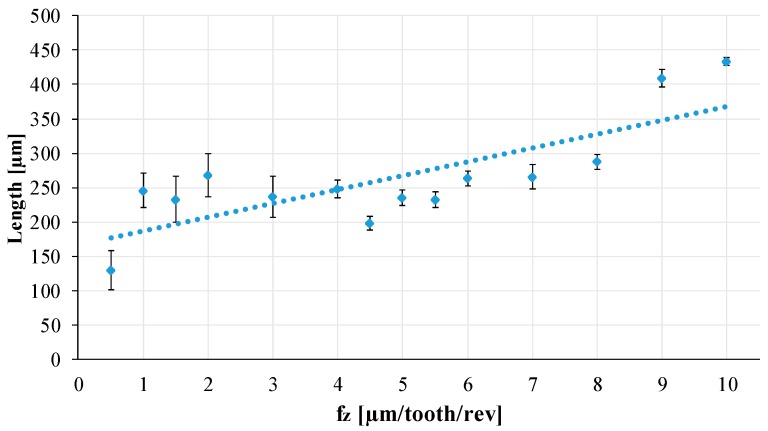
Chip length analysis. fz: feed per tooth.

The acquired images were used for the analysis and measurements using the Hirox Rh-2000 software, in particular, to measure the chip global length. A linearly increasing trend for the chip length, as fz increases, can be observed in Figure 11 that shows the average value for the chip length measurement and the standard deviation.

### 4.4. Microhardness

**Figure 12:**
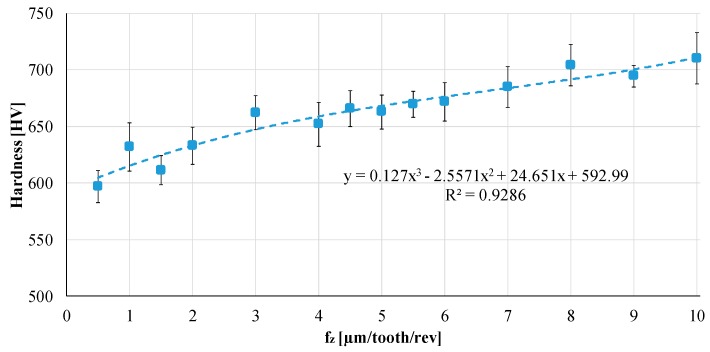
Microhardness: increasing trend when fz (feed per tooth) increases.

Finally, microhardness was measured on the microchannels. Figure 12 shows the results in terms of the mean (the final value is the average of 20 measured points on each channel) and standard deviations. According to this graph, an increasing trend in the microhardness with an increase in fz could be observed. In particular, the hardness values measured on the microchannels ranged from 597 HV (fz = 0.5 µm/tooth/rev) to 710 HV (fz = 10 µm/tooth/rev). The microhardness trend for intermediate fz values could be approximated by a third order polynomial trend line characterized by an R^2^ value of 0.928. These results suggest that a hardening phenomenon occurred when the sample was machined, and the increasing hardness is directly linked to the fz value set for the micro-milling operation.

## 5. Conclusions

This research deepens the cutting mechanisms of an additively manufactured cobalt-chromium-molybdenum (Co-Cr-Mo) alloy machined by micro-milling. The main aim of the work was to provide some solid basis to fill the lack of scientific research on the micro-milling of a metallic material produced by AM. Therefore, an ASTM F75 sample was produced with the SLM technique and then micromachined. 

Several experimental tests were performed. Firstly, the layered effect was investigated through preliminary micro-milling tests. Microchannels with different axial depths of cut were made, and the cutting force was monitored. Results show that the cutting force is not affected by the axial depth of cut, demonstrating there is no relevant layered effect. Other tests were made to measure and analyze roughness (Ra and Sa) and microhardness. Then, also the chip morphology was investigated. Finally, further forces analyses were performed. 

Results show that very low surface roughness can be achieved with the micro-milling operation, but no correlation between roughness (both Ra and Sa) and fz was observed. With regard to the chip morphology, the chip global length exhibited a linearly increasing trend as fz increases. Microhardness analysis highlighted a hardening phenomenon that occurred during the micro-milling operation. In particular, the increase in hardness was directly linked to the fz. Finally, forces analysis enabled the determination of the MUCT value for the SLM fabricated Co-Cr-Mo material. 

## Figures and Tables

**Table 1:** ASTM F75 main properties [24].

Properties	Units	ASTM F75
Mechanical properties		
Elastic Modulus	GPa	210 ÷ 250
Elongation at Break	%	12
Poisson Ratio	-	0.29
Shear Modulus	GPa	82 ÷ 98
Ultimate Tensile Strength	MPa	780 ÷ 1280
Yield Tensile Strength	MPa	480 ÷ 840
Thermal properties		
Specific Heat Capacity	J/Kg-K	450
Thermal Conductivity	W/m-K	13
Thermal Expansion	µm/m-K	12

**Table 2:** Micro end mill PMT TR-2-0020-S main features.

Cutting Diameter	End Cut	Helix	Flute Length	Flutes	Shank Diameter	Overall Length
508 µm	Square End	762 µm	1524 µm	2	1/8	38.1 mm

**Table 3:** SECO 905004-MEGA-T tool geometry.

Cutting Diameter	a_p_ Max	Total Length	Helix Angle	Flutes	r_ε_
0.4 mm	0.6 mm	40 mm	20°	2	5 ± 0.1 µm

a_p_: depth of cut; r_ε_: cutting edge radius.

**Table 4:** Feed per tooth (fz) levels for microchannels realization.

fz [µm/tooth/rev]													
0.5	1	1.5	2	3	4	4.5	5	5.5	6	7	8	9	10

## Supplementary Materials

The following are available online at https://www.mdpi.com/1996-1944/12/13/2208/s1, Figure S1: Table 1. ASTM F75 composition requirements, Figure S2: Microstructural observation: (a) BSE image of the sample; (b) crystal orientation map of the crystal structure, Figure S3: Burr—2D and 3D view: (a) a_p_ = 10 μm; (b) a_p_ = 25 μm; (c) a_p_ = 40 μm
