# Supplementary material for: An Experimental Study on Micro-Milling of a Medical Grade Co-Cr-Mo Alloy Produced by Selective Laser Melting
Source: Materials (Basel). 2019 Jul 9;12(13):2208. doi: 10.3390/ma12132208 (PMC6651660; doi:10.3390/ma12132208)
Supplement: Supplementary file 1 [file materials-12-02208-s001.pdf]

An Experimental Study on Micromilling of a Medical Grade Co-Cr-Mo Alloy Produced by Selective Laser Melting

Gabriele Allegri [1], Alessandro Colpani [1], Paola Serena Ginestra[1] and Aldo Attanasio [1,*]

Department of Mechanical and Industrial Engineering, University of Brescia, Via Branze 38, 25123, Brescia (BS), Italy

* Correspondence: aldo.attanasio@unibs.it; Tel.: +39-0303715584

Supplementary material

**Cr**	**27-30 %**	**Si**	**0-1 %**	**N**	**0-0.25 %**
**Mo**	5-7 %	**Mn**	0-1 %	**Al**	0-0.1 %
**Ni**	0-0.5 %	**W**	0-0.2 %	**Ti**	0-0.1 %
**Fe**	0-0.75 %	**P**	0-0.02 %	**B**	0-0.01 %
**C**	0-0.35 %	**S**	0-0.01 %	**Co**	Balance

Figure S1. Table 1. ASTM F75 composition requirements [30].

After producing the sample, microstructural characterization was performed using Scanning Electron Microscopy with a BSE (backscatter electron) detector that is sensitive to phase and crystal orientation. Figure S2a provides a BSE image and figure S2b the crystal orientation map. The picture was obtained from the x-y plane of the sample on the top surface.

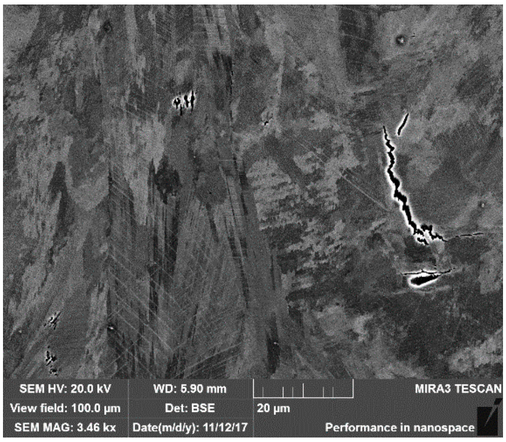

(a)

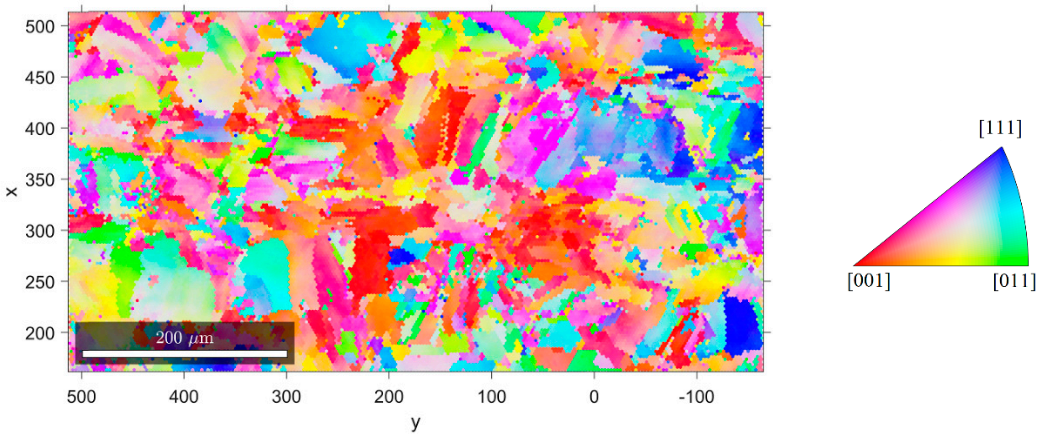

(b)

Figure S2. Microstructural observation: (a) BSE image of the sample; (b) crystal orientation map of the crystal structure.

Moreover, during this phase the influence of a_p on the burr formation was evaluated. Figure 9 shows that the axial depth of cut does not affect the burr formation. Burr formation in micromilling the SLM sample was evaluated since this phenomenon affects the final part quality in terms of accuracy and assembly functionality. For this analysis, 8 microchannels in the X direction and 8 in the Y direction were produced by changing the axial depth of cut (ap) that ranges from 5 μm to 40 μm (figure S3). Machining parameters were the same as those used for the layer effect test.

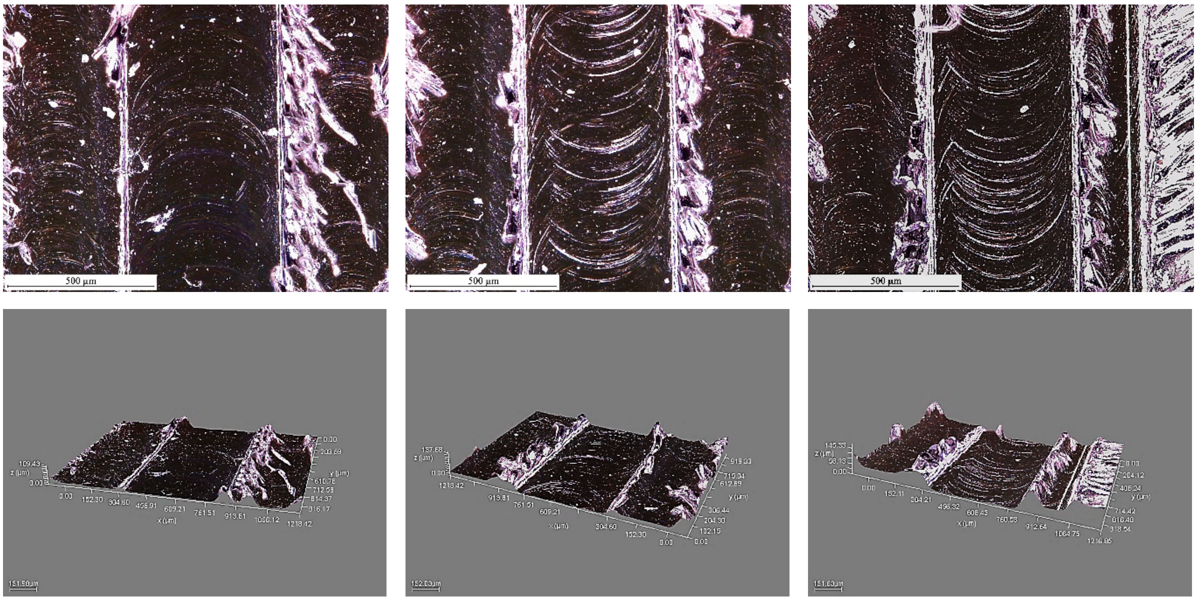

(a) (b) (c)

Figure S3. Burr—2D and 3D view: (a) a_p = 10 μm; (b) a_p = 25 μm; (c) a_p = 40 μm.

© 2019 by the authors. Submitted for possible open access publication under the terms and conditions of the Creative Commons Attribution (CC BY) license (http://creativecommons.org/licenses/by/4.0/).
